# TLR4 May Be Involved in the Regulation of Colonic Mucosal Microbiota by Vitamin A

**DOI:** 10.3389/fmicb.2019.00268

**Published:** 2019-02-22

**Authors:** Lu Xiao, Baolin Chen, Di Feng, Ting Yang, Tingyu Li, Jie Chen

**Affiliations:** Children’s Nutrition Research Center, Children’s Hospital of Chongqing Medical University, Ministry of Education Key Laboratory of Child Development and Disorders, China International Science and Technology Cooperation Base of Child Development and Critical Disorders, Chongqing, China

**Keywords:** toll-like receptor 4 (TLR4), vitamin A normal (VAN), vitamin A deficiency (VAD), intestinal mucosa-associated microbiota, intestinal innate immunity

## Abstract

**Objectives:** To investigate the specific role of Toll-like receptor 4 (TLR4) in the regulation of the intestinal mucosa-associated microbiota by vitamin A (VA).

**Methods:** Both TLR4^-/-^ (knockout, KO) and wild-type (WT) female mice were randomly fed a VA normal (VAN) or VA deficient (VAD) diet for 4 weeks to establish the following four mouse model groups: TLR4^-/-^ mice fed a VAN diet (KO VAN), TLR4^-/-^ mice fed a VAD diet (KO VAD), WT mice fed a VAN diet (WT VAN), and WT mice fed a VAD diet (WT VAD). Then, the mice from each experimental group were mated with male mice with the same genetic background. The pups in the KO VAD and WT VAD groups were subsequently fed the VAD diet after weaning, while the pups in the KO VAN and WT VAN groups were fed the VAN diet continuously after weaning. The serum retinol levels of 7-week-old offspring were determined using high-performance liquid chromatography, and colons were collected from mice in each group and analyzed via 16S rRNA gene sequencing using an Illumina MiSeq platform to characterize the overall microbiota of the samples.

**Results:** The abundance and evenness of the colon mucosa-associated microbiota were unaffected by dietary VA and TLR4 KO. VAD decreased the abundance of *Anaerotruncus* (*Firmicutes*), *Oscillibacter* (*Firmicutes*), *Lachnospiraceae* _*NK4A136* _*group* (*Firmicutes*) and *Mucispirillum* (*Deferribacteres*) and increased the abundance of *Parasutterella* (*Proteobacteria*). TLR4 KO decreased the abundance of *Bacteroides* (*Bacteroidetes*) and *Alloprevotella* (*Bacteroidetes*). However, the abundance of *Allobaculum* (*Firmicutes*), *Ruminiclostridium_9* (*Firmicutes*), *Alistipes* (*Bacteroidetes*), and *Rikenellaceae_RC9* (*Bacteroidetes*) impacted the interaction between VA and TLR4.

**Conclusion:** TLR4 may play a pivotal role in regulation of the intestinal mucosa-associated microbiota by VA to maintain the intestinal microecology.

## Introduction

Vitamin A is an essential fat-soluble vitamin that maintains normal growth and development, participates in the immune response, promotes reproduction, and maintains visual function (Stephensen, 2001; Clagett-Dame and DeLuca, 2002). VAD remains a significant public health concern in many regions of the world (World Health Organization [WHO], 1995). Children experiencing VAD are especially prone to gastrointestinal (GI) tract infections (Thornton et al., 2014). Studies by us and others have shown that VAD can decrease gut integrity and impact the immune response of the GI tract in humans and animals (Quadro et al., 2000; Liu et al., 2014). The diversity and balance of the gut microbiota is important for maintaining the normal biological barrier function of the intestine (Topping and Clifton, 2001; Round et al., 2010). Recent studies have shown that the VA nutritional status can affect the total amount of bacteria in the GI tract and alter the intestinal microflora (Amit-Romach et al., 2009). Our preliminary study found that VAD impacts the structural segregation of the gut microbiota in children with persistent diarrhea (Lv et al., 2016).

Toll-like receptors (TLRs) are membrane-anchored proteins that are expressed on immune cells and enterocytes (Takeda et al., 2003). TLRs act as pathogen recognition receptors (PRRs), identifying microbe-associated molecular patterns (MAMPs) to activate specific signaling pathways (Frosali et al., 2015). A total of 10 TLRs are expressed in humans, and TLR4 is the best characterized PRR. Recognition of MAMPs by TLR4 is involved in protective innate immune response mechanisms against bacterial invasion (Furuta et al., 2006). In addition, TLR4^-/-^ mice exhibited a striking reduction in acute inflammatory cells, impaired epithelial cell proliferation and marked bacterial translocation during injury compared with WT mice (Fukata et al., 2005). Moreover, mouse epithelial cells overexpressing the TLR4 signaling pathway exhibited increased bacterial density in the colonic mucosa and increased bacterial translocation (Dheer et al., 2016). In our previous study, we confirmed that RARβ enhanced ZO-2 expression by regulating TLR4 to improve intestinal epithelial barrier function both *in vivo* and *in vitro* (Li et al., 2017). However, the role of TLR4 in regulation of the gut microbiota by VA is unclear.

Therefore, the purpose of this study was to determine the effect of TLR4 on the intestinal mucosal microbiota associated with VA nutritional levels. In the present study, TLR4^-/-^ and WT mice were acquired to establish both VAN and VAD mouse models. 16S rRNA deep sequencing was used to examine the distribution and structural characteristics of the intestinal mucosa-associated microbiota.

## Materials and Methods

### Animals, Diets and Sample Collection

This study was approved by the Animal Experimentation Ethics Committee of Chongqing Medical University (Chongqing, China) and was conducted in accordance with the guidelines of the Animal Care Committee of Chongqing Medical University. TLR4^-/-^ (knockout, KO) and WT mice obtained from Jackson laboratories (Maine, United States) were purchased from the Model Animal Research Center of Nanjing University (MARC). The TLR4^-/-^ mouse strain was C57BL/10ScNJNju, which is based on the C57BL/10JNju mouse strain (WT). The mice were housed in the same room with a constant airflow system, controlled temperature (22–24°C), and a 12-h light/dark cycle. The VAN and VAD animal models were constructed according to methods described previously (Liu et al., 2014). Half of the female KO and WT mice (3 weeks of age), which were randomly selected, were fed a VAD-inducing diet comprising 400 IU/kg VA for 4 weeks to establish a TLR4^-/-^ mouse model with VAD (KO VAD) and a WT mouse model with VAD (WT VAD), and the other half received a VAN diet containing 6,500 IU/kg VA for 4 weeks to establish a VAN TLR4^-/-^ mouse model (KO VAN) and a VAN WT mouse model (WT VAN). Then, the (female) mice from each experimental group were mated with the corresponding male mice with the same genetic background. Pregnant mice were fed either the VAD or VAN diet during both gestation and lactation to maintain stable serum retinol levels. Once the pups had weaned, their mothers were sacrificed, and blood was collected from the eyeballs. The serum retinol levels of the maternal VAN mice increased to 1.05 μmol/L, and those of the maternal VAD mice decreased to 0.7 μmol/L. The offspring were used for subsequent experiments. The pups in the KO VAD and WT VAD groups were subsequently fed the VAD diet continuously for 4 weeks, while the pups in the KO VAN and WT VAN groups were fed the VAN diet continuously for the same time period. Next, the mice were sacrificed, and blood was immediately harvested from the eyeball. The colons were extracted from the mice in each group, and after cleaning with 0.01 M PBS, the colons were stored at -80°C until further study.

### Serum Retinol Detection

The serum retinol levels in the collected mouse blood were determined using HPLC. VA standard curve preparation and testing methods were modified slightly following methods described previously (Li et al., 2017), and VA standard compound was purchased from Sigma (R7632, United States). Briefly, 200 μL of serum was deproteinized with the same volume of anhydrous ethanol. Then, 1000 μL of hexane was used to extract the retinol from the serum, and the hexane was evaporated using nitrogen gas. The retinol residue was dissolved in 100 μL of the mobile phase mixture (methanol:water = 97:3). Finally, the prepared sample was measured using an HPLC apparatus (DGU-20As, Shimadzu Corporation, Japan). The retinoids were separated by chromatography on an analytical column (Hypersil phenyl 120 A 5 mm, 250 mm × 4.6 mm, Phenomenex, United States) via gradient elution of the mobile phase in a liquid chromatograph equipped with a 315-nm ultraviolet photodiode array detector.

### DNA Extraction and PCR Amplification

Microbial DNA was extracted from colon samples using an OMEGA DNA Kit (Omega Bio-Tek, United States) according to the manufacturer’s protocol. The final concentration of the purified DNA was determined with a NanoDrop 2000 UV-vis spectrophotometer (Thermo Fisher Scientific, Wilmington, DE, United States), and DNA quality was checked via 1% agarose gel electrophoresis. The V3-V4 hypervariable regions of the bacterial 16S rRNA gene were amplified with the primers 338F (5′-ACTCCTACGGGAGGCAGCAG-3′) and 806R (5′-GGACTACHVGGGTWTCTAAT-3′) (Xu et al., 2016) using a PCR thermocycler system (GeneAmp 9700, ABI, United States). The PCRs were conducted using the following program: 3 min of denaturation at 95°C; 27 cycles of 30 s at 95°C, 30 s for annealing at 55°C, and 45 s for elongation at 72°C; and a final extension at 72°C for 10 min. The PCRs were performed in triplicate in 20-μL mixtures containing 4 μL of 5 × FastPfu buffer, 2 μL of 2.5 mM dNTPs, 0.8 μL of each primer (5 μM), 0.4 μL of FastPfu polymerase and 10 ng of template DNA. The resulting PCR products were extracted from a 2% agarose gel, further purified using an AxyPrep DNA Gel Extraction Kit (Axygen Biosciences, Union City, CA, United States) and quantified using QuantiFluor^TM^-ST (Promega, United States) according to the manufacturer’s protocol.

### Illumina MiSeq Sequencing

Colon samples were collected from 40 mice from 4 groups: KO VAN group, KO VAD group, WT VAN group, and WT VAD group, with 10 samples per group. After DNA extraction and PCR amplification, the target band size and concentration of the samples to be sequenced were correct. Purified amplicons were pooled in equimolar amounts, and paired-end (2 × 300) sequencing was performed on an Illumina MiSeq platform (TruSeq^TM^ DNA Sample Prep Kit, United States) according to the standard protocols recommended by Majorbio Bio-Pharm Technology Co., Ltd. (Shanghai, China). The raw reads were deposited into the NCBI Sequence Read Archive (SRA) database (Accession Number: SRP: 158355).

### Processing of Sequencing Data

Raw fastq files were demultiplexed, quality-filtered with Trimmomatic and merged using FLASH with the following criteria: (i) The reads were truncated at any site that received an average quality score <20 over a 50-bp sliding window. (ii) Primers were exactly matched, allowing 2-nucleotide mismatches, and reads containing ambiguous bases were removed. (iii) Sequences with overlaps longer than 10 bp were merged at the overlap sequence.

Operational taxonomic units were clustered with a 97% similarity cutoff using UPARSE (version 7.1^1^ ), and chimeric sequences were identified and removed using UCHIME. The taxonomy of each 16S rRNA gene sequence was analyzed using the RDP Classifier algorithm^2^ compared against the Silva (SSU128) 16S rRNA database using a confidence threshold of 70%.

### Microbial Analysis

Sets of sequences with 97% identity were clustered into OTUs using USEARCH (version 7.0^3^). OTUs that reached 97% similarity levels were used for community richness (Chao, ACE), community diversity (Shannon, Simpson), and rarefaction curve analyses. The β-diversity was estimated by computing unweighted UniFrac distances and visualized with principal coordinate analysis. To effectively distinguish between the four groups, a partial least squares discriminant analysis (PLS-DA) was conducted. In addition, linear discriminant analysis (LDA) effect size (LEfSe) was determined using LEfSe software to determine the community or species that most influenced the group division. After features that were significantly different at various bacterial taxonomic levels were identified by LEfSe, the nonparametric factorial Kruskal-Wallis (KW) sum-rank test and LDA were performed to determine whether these features were consistent with the expected behaviors of the different bacterial taxonomic levels; genera with LDA scores greater than three were defined as having a significant impact on the group.

### Statistical Analyses

All data were obtained from ten biological replicates and are presented as the mean ± SEM. Significant differences were calculated via two-way analysis of variance (ANOVA) with a Bonferroni *post hoc* test using the GraphPad Prism version 5.0 software package. The interaction between the effect of the different VA nutrition levels and the effect of TLR4 deletion was investigated with a Bonferroni *post hoc* test. When there was a statistically significant interaction, all the experimental groups were compared using a Bonferroni *post hoc* test. However, when no interaction was observed, the effect of the different VA nutrition levels or TLR4 deletion was assessed using Student’s *t*-test. Only the relevant comparisons of the combined groups are presented in the Results section. Significance was accepted at *P* < 0.05.

## Results

### The VAD Diet Decreased Serum Retinol Levels in Mice

**FIGURE 1:**
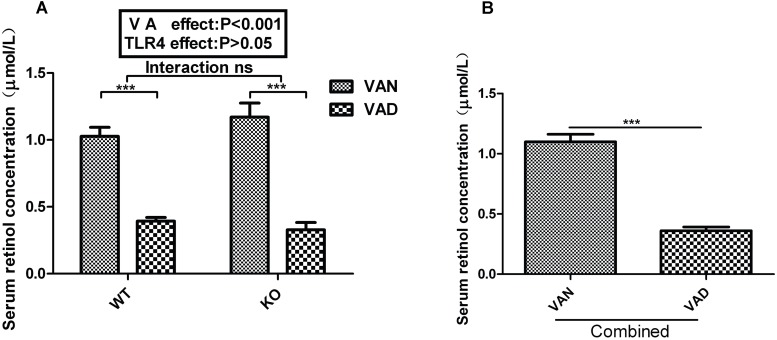
Effects of different VA nutritional levels and TLR4 deletion on the serum retinol levels of seven-week-old offspring mice. **(A)** Changes in the serum retinol levels of the WT and TLR4^-/-^ mice fed VAN or VAD diets (*n* = 10 for each group). **(B)** The main effect of VA, independent of TLR4^-/-^ challenge, on the differences in serum retinol levels between the combined VAN and VAD groups (*n* = 20). The values are the means ± SEMs; “Interaction” indicates an effect of the different VA nutritional levels in the TLR4 knockout vs. WT mice; ^∗∗∗^*P* < 0.001. VAN, vitamin A normal; VAD, vitamin A deficiency. WT VAN refers to WT mice fed a VAN diet; WT VAD refers to WT mice fed a VAD diet; KO VAN refers to TLR4^-/-^ mice fed a VAN diet; KO VAD refers to TLR4^-/-^ mice fed a VAD diet.

To explore whether TLR4 participates in regulation of intestinal microbial homeostasis by VA, we established VAN and VAD mouse models in both TLR4^-/-^ (KO VAN and KO VAD) and WT mice (WT VAN and WT VAD). As shown in Figure 1A, the serum retinol levels of offspring in WT mice fed the VAD diet (0.393 ± 0.027 μmol/L) were significantly lower than those in WT mice fed the VAN diet (1.027 ± 0.067 μmol/L) (*P* < 0.001). Similar results were observed for the TLR4^-/-^ mice, and the serum retinol levels were significantly repressed in the KO VAD group (0.328 ± 0.054 μmol/L) compared with those in the KO VAN group (1.171 ± 0.104 μmol/L). However, there was no significant interaction between the effects of VA and TLR4, determined using two-way ANOVA with a Bonferroni *post hoc* test, but differences in VA nutritional levels had an effect on the serum retinol levels (*P* < 0.001, Figure 1A). After combining the data for the WT and KO groups, we found that the serum retinol levels in the combined VAD group were markedly lower than those in the combined VAN group (*P* < 0.001) (Figure 1B). The above data demonstrate that the VAD diet was an important factor associated with the decreased serum retinol levels in mice, providing us with a solid foundation for subsequent studies.

### Rarefaction Curves and Alpha Diversity Index

**FIGURE 2:**
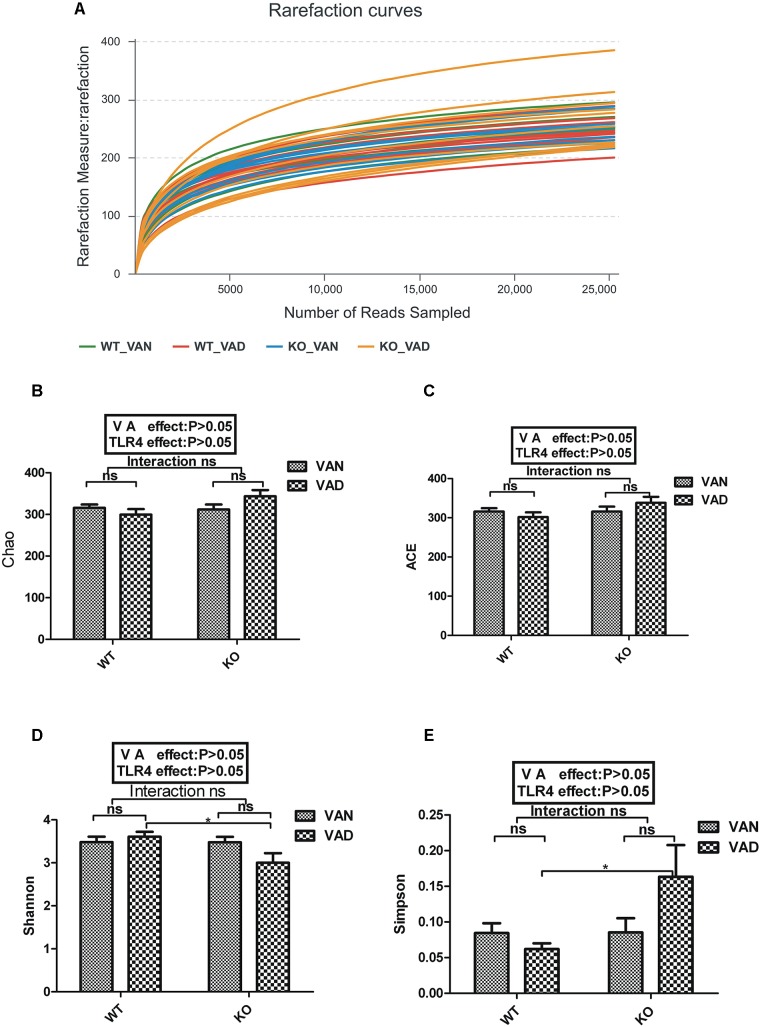
Rarefaction curves and α diversity analysis for WT and TLR4^-/-^ mice fed VAN or VAD diets. **(A)** Rarefaction curves were calculated for OTUs with 97% identity in the gut microbiota in the WT VAN, WT VAD, KO VAN, and KO VAD groups (*n* = 10). The green curves represent the WT VAN group; red curves represent the WT VAD group; blue curves represent the KO VAN group; yellow curves represent the KO VAD group (*n* = 10). Comparison of **(B)** Chao index, **(C)** ACE index, **(D)** Shannon index and **(E)** Simpson index among the four groups (*n* = 10). Mean ± SEM; ns. = not significant. “Interaction” indicates an effect of the different VA nutritional levels in the TLR4 knockout vs. WT mice; ^∗^*P* < 0.05. VAN, vitamin A normal; VAD, vitamin A deficiency. WT VAN refers to WT mice fed a VAN diet; WT VAD refers to WT mice fed a VAD diet; KO VAN refers to TLR4^-/-^ mice fed a VAN diet; KO VAD refers to TLR4^-/-^ mice fed a VAD diet.

After optimization, a total of 1,487,466 high-quality sequences were obtained from 40 samples, and there were 37,187 high-quality sequences per sample on average according to MiSeq sequencing. We acquired numerous OTUs from valid sequences that exhibited 97% similarity for further statistical analyses. Along with an increase in the number of reads, the rarefaction curves for all the samples shown in Figure 2A exhibited smooth increasing trends and approached saturation plateaus, demonstrating that the sequencing data volume acquired was suitable for the present study.

In general, Chao and ACE diversity indexes reflect the richness of the microbiota, while Shannon and Simpson diversity indexes are considered to be indicators of colony richness and evenness. After two-way ANOVA with a *post hoc* test, no significant interaction was found between the effects of VAD and TLR4 KO on the Chao, ACE, Shannon and Simpson diversity indexes (Figure 2B–E). Furthermore, these four indexes were not affected by either VAD or TLR4 KO, even though the Simpson index of the KO VAD group was slightly higher than that of the WT VAD group (*P* < 0.05, Figure 2E) and the Shannon index of the KO VAD group was significantly decreased when compared with that of the WT VAD group (*P* < 0.05, Figure 2D). The above data demonstrate that the abundance and evenness of the colonic mucosa-associated microflora were unaffected by dietary VA and TLR4 KO.

### Different VA Levels in the Diet May Affect the Microbial Community Structure of the Colonic Mucosa in Both the TLR4 KO and WT Mice

**FIGURE 3:**
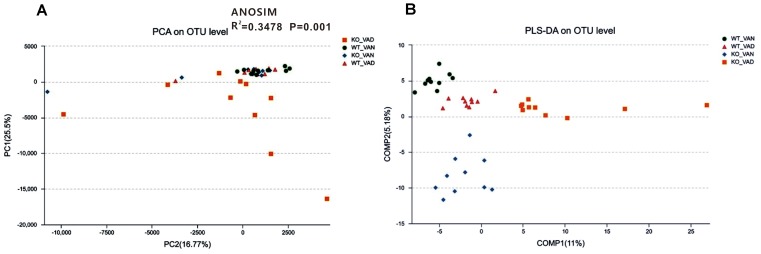
PCA and PLS-DA of samples from the TLR4^-/-^ and WT mice fed VAN or VAD diets. **(A)** PCA scores were plotted based on the relative abundance of the OTUs (*n* = 10). **(B)** PLS-DA was plotted based on the unweighted UniFrac distance metrics (*n* = 10). The green circles represent the WT VAN group; red triangles represent the WT VAD group; blue diamonds represent the KO VAN group; orange squares represent the KO VAD group (*n* = 10). VAN, vitamin A normal; VAD, vitamin A deficiency. WT VAN refers to WT mice fed a VAN diet; WT VAD refers to WT mice fed a VAD diet; KO VAN refers to TLR4^-/-^ mice fed a VAN diet; KO VAD refers to TLR4^-/-^ mice fed a VAD diet.

To further evaluate structural differences in the microbial communities among the four groups, an unweighted UniFrac distance matrix was calculated based on the OTUs of each sample. Figure 3A shows that samples from the WT VAN, WT VAD, and KO VAN groups were relatively concentrated compared with the KO VAD group samples. Principle component analysis (PCA) revealed a separation of the TLR4^-/-^ and WT mice fed VAN or VAD diets based on the first two principal component (PC) scores, accounting for 25.5 and 16.77% of explained variances (Figure 3A). Meanwhile, ANOSIM analysis showed that the difference among the four groups was significantly greater than the difference within the group (*R*^2^ = 0.3478, *P* = 0.001, Figure 3A), indicating that our grouping was meaningful. The results of the subsequent PLS-DA are shown in Figure 3B, and the variance among the KO VAD group samples was greater than that among samples from the other three groups. These data suggest that there may be differences in the distribution of the colonic mucosal microbiota due to the different levels of VA in the diet and due to TLR4 deletion.

### Both VA and TLR4 Are Involved in the Community Abundance of the Colonic Mucosal Microbiota at the Phylum Level

**FIGURE 4:**
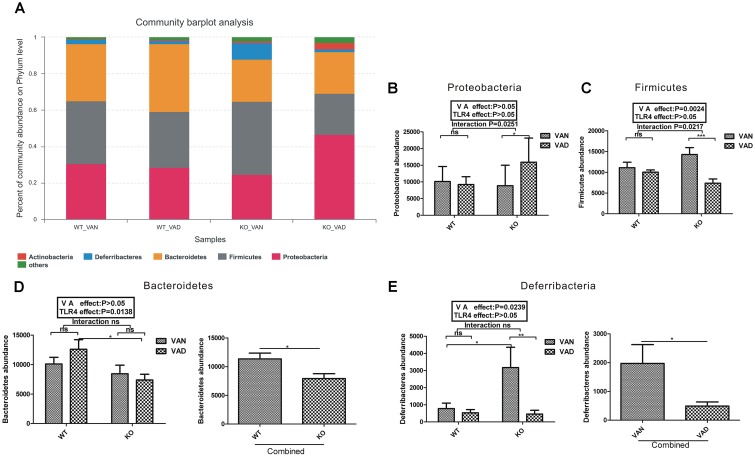
The relative abundances of bacterial phyla in the colonic mucosa of the TLR4^-/-^ and WT mice fed the VAN or VAD diet. **(A)** The dominant bacterial phyla with relative abundances greater than 1% in the four groups (*n* = 10). The combined effects of the different VA nutritional levels and the TLR4 deletion on the relative abundance of **(B)** *Proteobacteria* and **(C)** *Firmicutes* determined by two-way analysis of variance with a Bonferroni *post hoc* test (*n* = 10). **(D)** The main effect of TLR4, independent of VA nutritional level, on the relative abundance of *Bacteroidetes* in the four groups (*n* = 10). **(E)** The main effect of VA, independent of TLR4 deletion, on the relative abundance of *Deferribacteres* in the four groups (*n* = 10). Mean ± SEM; ns. = not significant. “Interaction” indicates an effect of the different VA nutritional levels in the TLR4 knockout vs. WT mice; ^∗^*P* < 0.05, ^∗∗^*P* < 0.01, and ^∗∗∗^*P <* 0.001. VAN, vitamin A normal; VAD, vitamin A deficiency. WT VAN refers to WT mice fed a VAN diet; WT VAD refers to WT mice fed a VAD diet; KO VAN refers to TLR4^-/-^ mice fed a VAN diet; KO VAD refers to TLR4^-/-^ mice fed a VAD diet.

Figure 4A shows the composition of the dominant microflora with a relative abundance of more than 1% at the phylum level in the TLR4 KO and WT mice fed the VAN or VAD diet. In the WT VAN group, the predominant phyla were *Firmicutes* (34.47%), *Bacteroidetes* (31.34%), *Proteobacteria* (30.62%), and *Deferribacteres* (2.372%); however, the predominant phyla in the WT VAD group were *Bacteroidetes* (37.33%), *Firmicutes* (30.91%), *Proteobacteria* (28.38%), and *Deferribacteres* (1.718%). The most abundant phyla in the KO VAN group, in decreasing order, were *Firmicutes* (40%), *Proteobacteria* (24.66%), *Bacteroidetes* (23.42%), and *Deferribacteres* (8.928%). Notably, the percentage of *Actinobacteria* was highest in the KO VAD group compared with that in the other three groups, and the most abundant phyla, in decreasing order, in the KO VAD group were *Proteobacteria* (46.58%), *Bacteroidetes* (22.92%), *Firmicutes* (22.69%), *Actinobacteria* (3.644%), and *Deferribacteres* (1.357%).

As shown in Figure 4B, although VA and TLR4 had no effect on the abundance of *Proteobacteria* (*P* > 0.05), the *P*-value of the interaction between the VA and TLR4^-/-^ challenges was 0.0251 after two-way ANOVA with a Bonferroni *post hoc* test. The *Firmicutes* abundance in the VAD group was lower than that in the VAN group in the TLR4^-/-^ mice (Figure 4C). The VA nutritional level had significant effects on the *Firmicutes* abundance (*P* = 0.0024), and the *P*-value of the interaction between the VA and TLR4^-/-^ challenges for the *Firmicutes* abundance was 0.0217 according to the *post hoc* test (Figure 4C). However, for the abundance of *Bacteroidetes* and *Deferribacteres*, no significant interaction was observed between the VA and TLR4^-/-^ challenges, determined using two-way ANOVA (Figure 4D,E). After combining the VAN and VAD groups, the *Bacteroidetes* abundance in the combined KO group was significantly lower than that in the combined WT group (*P* < 0.05, Figure 4D). The *Deferribacteres* abundance in the combined VAD group was significantly lower than that in the combined VAN group (*P* < 0.05, Figure 4E). Based on these data, VA and TLR4 have interactive effects on the abundance of *Proteobacteria* and *Firmicutes*; the *Bacteroides* abundance was affected by TLR4 and that of *Deferribacteres* was affected by VA.

### VA and TLR4 Altered the Community Structure of the Colonic Microbiota at the Genus Level

**FIGURE 5:**
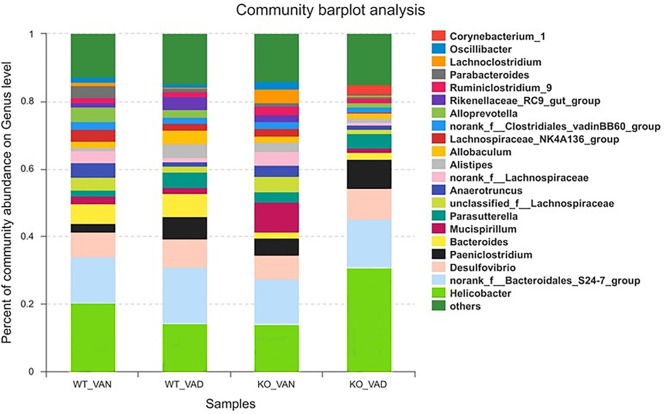
The dominant bacterial genera with relative abundances greater than 2% in the colonic mucosa of TLR4^-/-^ and WT mice fed the VAN or VAD diet (*n* = 10). VAN, vitamin A normal; VAD, vitamin A deficiency. WT VAN refers to WT mice fed a VAN diet; WT VAD refers to WT mice fed a VAD diet; KO VAN refers to TLR4^-/-^ mice fed a VAN diet; KO VAD refers to TLR4^-/-^ mice fed a VAD diet.

To further understand the effect of VA and TLR4 on colonic bacteria, we analyzed the community structure at the genus level in the four groups. Figure 5 shows the composition of the dominant microflora with relative abundances greater than 2% at the genus level in the TLR4^-/-^ and WT mice fed the VAN or VAD diet. We noticed that the percentage of *Helicobacter* in the KO VAD group was highest compared with the percentage in the other three groups.

**FIGURE 6:**
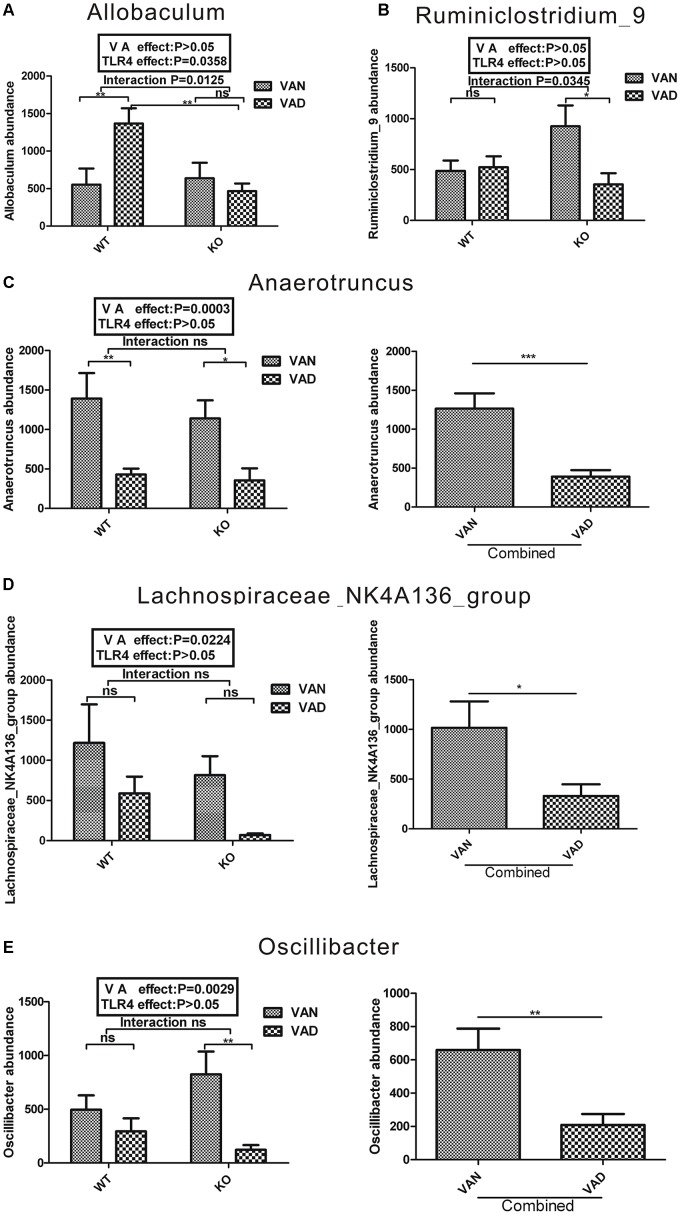
Two-way ANOVA of the five genera with relative abundances greater than 2% from the phylum *Firmicutes* in the colonic mucosa of TLR4^-/-^ and WT mice fed the VAN or VAD diet. The combined effects of the different VA nutritional levels and the TLR4 deletion on the relative abundance of **(A)** *Proteobacteria* and **(B)** *Ruminiclostridium_9* (*n* = 10). The main effect of VA, independent of TLR4 deletion, on the relative abundance of **(C)** *Anaerotruncus*, **(D)** *Lachnospiraceae_NK4A136_group* and **(E)** *Oscillibacter* in the four groups (*n* = 10). Mean ± SEM; ns. = not significant. “Interaction” indicates an effect of the different VA nutritional levels in the TLR4 knockout vs. WT mice; ^∗^*P* < 0.05, ^∗∗^*P* < 0.01, and ^∗∗∗^*P <* 0.001. VAN, vitamin A normal; VAD, vitamin A deficiency. WT VAN refers to WT mice fed a VAN diet; WT VAD refers to WT mice fed a VAD diet; KO VAN refers to TLR4^-/-^ mice fed a VAN diet; KO VAD refers to TLR4^-/-^ mice fed a VAD diet.

Two-way ANOVA was conducted for each strain, as shown in Figure 6, 7. There were five genera of the phylum *Firmicutes* that were affected by VA or both VA and TLR4. A significant interaction was observed between the effects of VA and TLR4 on the abundance of *Allobaculum* and *Ruminiclostridium_9* according to a Bonferroni *post hoc* analysis, and the *P*-values were 0.0125 and 0.0345, respectively (Figure 6A,B). On the other hand, the main effects of VA, independent of TLR4, were on the relative abundance of *Anaerotruncus* (*P* = 0.0003), *Lachnospiraceae* _*NK4A136* _*group* (*P* = 0.0224), and *Oscillibacter* (*P* = 0.0029) in the four groups (Figure 6C–E). After combining the WT and KO groups, the abundance of *Anaerotruncus* (*P* < 0.001), *Lachnospiraceae_NK4A136_group* (*P* < 0.05), and *Oscillibacter* (*P* < 0.01) was markedly decreased in the combined VAD group compared with that in the combined VAN group (Figure 6C–E).

**FIGURE 7:**
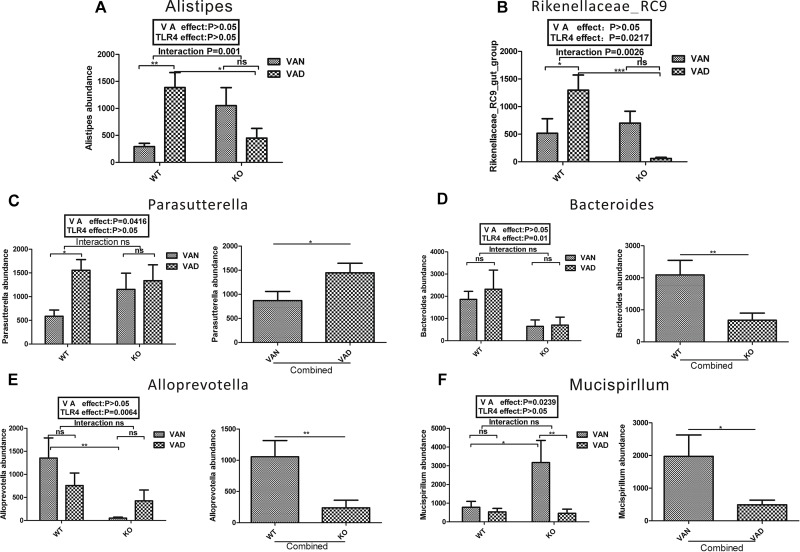
Two-way ANOVA of genera with relative abundances greater than 2% from the phyla *Proteobacteria*, *Bacteroidetes*, and *Deferribacteres* in the colonic mucosa of the TLR4^-/-^ and WT mice fed the VAN or VAD diet. The combined effects of the different VA nutritional levels and TLR4 deletion on the relative abundance of **(A)** *Alistipes* and **(B)** *Rikenellaceae_RC9* (*n* = 10). The main effect of VA, independent of TLR4 deletion, on the relative abundance of **(C)** *Parasutterella* and **(F)** *Mucispirillum* in the four groups (*n* = 10). The main effect of TLR4, independent of VA levels, on the relative abundance of **(D)** *Bacteroides* and **(E)** *Alloprevotella* among the four groups (*n* = 10). Mean ± SEM; ns. = not significant. “Interaction” indicates an effect of the different VA nutritional levels in the TLR4 knockout vs. WT mice; ^∗^*P* < 0.05, ^∗∗^*P* < 0.01, and ^∗∗∗^*P <* 0.001. VAN, vitamin A normal; VAD, vitamin A deficiency. WT VAN refers to WT mice fed a VAN diet; WT VAD refers to WT mice fed a VAD diet; KO VAN refers to TLR4^-/-^ mice fed a VAN diet; KO VAD refers to TLR4^-/-^ mice fed a VAD diet.

Vitamin A and TLR4 also impacted the community structure at the genus level within the phyla *Bacteroidetes, Proteobacteria, and Deferribacteres*. According to two-way ANOVA, there was a significant interaction between the effects of VA and TLR4 on the abundance of *Alistipes* and *Rikenellaceae_RC9* in the phylum *Bacteroidetes*, and the *P-*values were 0.001 and 0.0026, respectively (Figure 7A,B). However, no significant interaction was observed between the effects of VA and TLR4 on the abundance of *Bacteroides* (Figure 7D) and *Alloprevotella* (Figure 7E). After combining the VAN and VAD groups, the abundance of *Bacteroides* and *Alloprevotella* in the combined KO group was markedly lower than that in the combined WT group. TLR4 seems to be the main factor affecting the abundance of *Bacteroides* and *Alloprevotella* at the *Bacteroidetes* level. However, the *Parasutterella* abundance (*Proteobacteria*) was significantly increased in the combined VAD group compared with that in the combined VAN group, and the abundance of *Mucispirillum* (*Deferribacteres*) was significantly reduced in the combined VAD group compared with that in the combined VAN group.

Based on these data, both VA and TLR4 affected the abundance of *Allobaculum* (*Firmicutes*), *Ruminiclostridium_9* (*Firmicutes*)*, Alistipes* (*Bacteroidetes*), and *Rikenellaceae_RC9* (*Bacteroidetes*), while the abundance of *Anaerotruncus* (*Firmicutes*), *Lachnospiraceae_NK4A136_group* (*Firmicutes*), *Oscillibacter* (*Firmicutes*)*, Parasutterella* (*Proteobacteria*), and *Mucispirillum* (*Deferribacteres*) was mainly affected by VA, and that of *Bacteroides* (*Bacteroidetes*) and *Alloprevotella* (*Bacteroidetes*) was primarily affected by TLR4.

### Key Phylotypes in the TLR4^-/-^ and WT Mice With Different VA Levels

**FIGURE 8:**
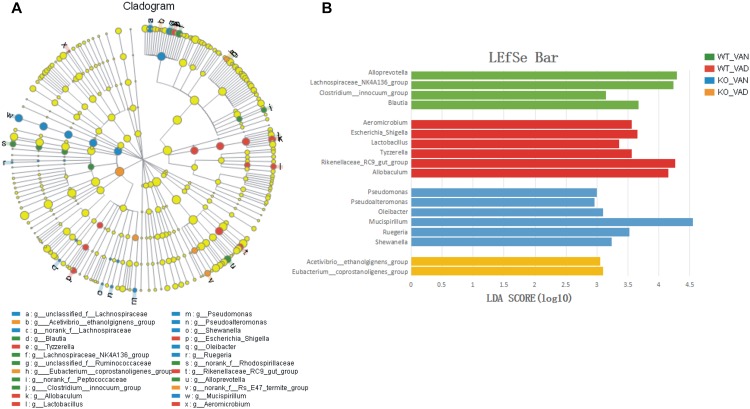
LEfSe analysis of the different structures of the colonic mucosal microbiota in the TLR4 KO and WT mice fed the VAN or VAD diet. **(A)** A cladogram of the statistical and biological differences in the colonic microbiota among the WT VAN, WT VAD, KO VAN, and KO VAD groups, which are shown by the color of the most abundant phylotypes (*n* = 10). **(B)** A histogram of the LDA scores for the most abundant phylotypes (*n* = 10). Mean ± SEM. VAN, vitamin A normal; VAD, vitamin A deficiency. WT VAN refers to WT mice fed a VAN diet; WT VAD refers to WT mice fed a VAD diet; KO VAN refers to TLR4^-/-^ mice fed a VAN diet; KO VAD refers to TLR4^-/-^ mice fed a VAD diet.

A metagenomic analysis approach (LEfSe) was used to identify the key phylotypes responsible for the differences among the TLR4 KO and WT mice fed the VAN or VAD diet. Figure 8 shows a comparison of the bacterial populations in the four groups at the genus level. The results indicated that the key genera in the WT VAN group were *Alloprevotella* (LDA = 4.34, *P* = 0.003751), *Lachnospiraceae_NK4A136_group* (LDA = 4.14, *P* = 0.000845), *Clostridium_innocuum_group* (LDA = 3.45, *P* = 0.000844), and *Blautia* (LDA = 3.68, *P* = 0.000227). In the WT VAD group, the key genera were *Aeromicrobium* (LDA = 3.81, *P* = 0.004725), *Escherichia_Shigella* (LDA = 3.63, *P* = 0.000193), *Lactobacillus* (LDA = 3.29, *P* = 0.007436), *Tyzzerella* (LDA = 3.58, *P* = 0.005026), *Rikenellaceae_RC9_gut_group* (LDA = 4.26, *P* = 0.000131), and *Allobaculum* (LDA = 4.13, *P* = 0.010607). *Oleibacter* (LDA = 3.19, *P <* 0.003641), *Pseudomonas* (LDA = 3.00, *P* = 0.000502), *Mucispirillum* (LDA = 4.54, *P =* 0.012631), *Ruegeria* (LDA = 3.51, *P* = 0.000837), *Shewanella* (LDA = 3.52, *P* = 0.023659), and *Pseudoalteromonas* (LDA = 3.07, *P <* 0.0001) were the key genera in the KO VAN group. However, only two genera played key roles in the KO VAD group: *Acetivibrio_ethanolgignens_group* (LDA = 3.07, *P* = 0.004929) and *Eubacterium_coprostanoligenes_group* (LDA = 3.15, *P* = 0.022025).

## Discussion

The GI tract harbors a complex community of bacteria in the mucosa, lumen and feces. Feces may primarily contain bacteria that are not adherent (Zoetendal et al., 2002), and the luminal microbiota is more variable than the mucosal microbiota (Li et al., 2015). Therefore, in our study, the mucosal bacteria may better reflect the actual intestinal microbiota (Amit-Romach et al., 2009). Some ecologists have noted that the Chao and ACE diversity indexes reflect the richness of the microbiota. The Shannon and Simpson diversity indexes are considered indicators of colony richness and evenness. The Simpson index is sensitive to dominant species, and the Shannon index is sensitive to rare species (Magurran, 1988). In the present study, TLR4 KO reduced the Shannon index and upregulated the Simpson index in the VAD group. TLR4 KO may decrease the abundance of rare species and increase the dominant intestinal mucosa-associated microbiota in VAD rats. However, VA had no effect on the Shannon and Simpson indexes in WT mice; therefore, these two indexes were not found to be affected by either VA or TLR4 after two-way ANOVA with a *post hoc* test. Although VA and TLR4 had no effect on the abundance and evenness of the colonic mucosa-associated microflora, the results of PCA and PLS-DA showed that VA and TLR4 affected the distribution and composition of the colonic mucosa-associated bacteria.

Amit-Romach showed that VAD led to a reduction in the proportion of *Lactobacillus* spp. and resulted in the appearance of pathogenic *Escherichia coli* strains among the mucosa-associated bacteria (Amit-Romach et al., 2009). Matthew C. found that acute VAD had an effect on bacterial community structure, leading to an increase in the abundance of *Bacteroides vulgatus* (Hibberd et al., 2017). Studies on changes in gut microbial diversity have shown varying results. However, in the present study, at the genus level, VAD decreased the abundance of *Anaerotruncus, Oscillibacter, Lachnospiraceae_NK4A136_group*, and *Mucispirillum* and increased the abundance of *Parasutterella.*

*Anaerotruncus* is a newly described bacterial genus isolated from human stool (Lawson et al., 2004). Currently, *Anaerotruncus colihominis* and *Anaerotruncus* *massiliensis* are the identified species of this genus (Lau et al., 2006). A. H. Togo isolated *Anaerotruncus massiliensis* from an obese patient after bariatric surgery (Togo et al., 2016). Although *Anaerotruncus* is not well known, studies have shown that *Anaerotruncus* species might be optimal probiotic strains because these species express enzymes that favor the production of butyrate (Polansky et al., 2015). Butyrates are important nutrients for cells lining the mammalian colon. As critical short-chain fatty acid derivatives that regulate colon homeostasis, butyrates participate in colon inflammation (Donohoe et al., 2011). In a study of human gut microflora, *Oscillibacter valericigenes* was identified in a significantly greater number of samples from healthy controls than from patients with Crohn’s disease (Man et al., 2011; Mondot et al., 2011). *Anaerotruncus* and *Oscillibacter* appear to play a positive role in maintaining intestinal immune homeostasis. *Lachnospiraceae* species are also associated with maintenance of gut health, and members of this family may protect against colon cancer in humans by producing butyric acid (Tap et al., 2009; Meehan and Beiko, 2014; Liu et al., 2017). Mice administered retinoic acid (RA) orally and then subjected to partial hepatectomy had higher levels of *Lachnospiraceae* than mice in the control group, which were not treated with RA (Liu et al., 2016). In the present study, VAD reduced the abundance of *Anaerotruncus* and *Oscillibacter* in the colonic mucosa, and the abundance of *Lachnospiraceae _NK4A136 _ group* was also lower in the combined VAD group than in the VAN group, while VAD upregulated the abundance of *Parasutterella*. Previously, the *Parasutterella* abundance was found to be increased and the *Lachnospiraceae* abundance was decreased in the submucosal tissues of patients with Crohn’s disease (Chiodini et al., 2015). In addition, the *Parasutterella* abundance was increased significantly in rats with hypertriglyceridemia-related acute necrotizing pancreatitis (Huang et al., 2017). These results suggest that VA may be involved in regulation of the intestinal mucosa-associated microbiota.

*Mucispirillum* is a core member of the laboratory mouse microbiota and can colonize the intestinal tract from the stomach to the colon; this genus is represented by a single species, namely, *Mucispirillum schaedleri* (Robertson et al., 2005). As part of the phylum *Deferribacteres*, *Mucispirillum* has been shown to be associated with both inflammatory markers (El Aidy et al., 2014) and active colitis in a T-bet^-/-^ Rag2^-/-^ mouse model (Berry et al., 2012; Rooks et al., 2014) and was even associated with *Citrobacter rodentium* infection (Hoffmann et al., 2009). A recent study showed that *M. schaedleri* possesses specialized systems to handle oxidative stress during inflammation (Loy et al., 2017). Interestingly, our data showed that VAD downregulated the abundance of *Mucispirillum*. We speculate that this downregulation may be a self-regulatory effect of the intestinal mucosa-associated microbiota.

Activation of TLRs by commensal microflora is critical for protection against gut injury (Rakoff-Nahoum et al., 2004; Fukata et al., 2005; Fukata et al., 2006). A large amount of research has indicated that TLR4 signaling affects the intestinal microbiota (Anitha et al., 2012). However, the effects of TLR4 on the intestinal mucosa-associated microbiota are complex and remain unclear. Our results suggest that TLR4 KO decreased the abundance of *Bacteroides* and *Alloprevotella*. *Bacteroides*, a commensal bacterium that colonizes the lower digestive tract, can strongly affect the host immune system (Swidsinski et al., 2009). A study by Erin B showed that *Bacteroides* species produce a capsular polysaccharide, polysaccharide A (PSA), to repress proinflammatory cytokines (Troy and Kasper, 2010). Many studies have implicated decreased levels of *Bacteroides* in the development of IBD (Zhou and Zhi, 2016). In our study, TLR4^-/-^ mice exhibited a defective immune response, which may be associated with the marked reduction in *Bacteroides* abundance. *Alloprevotella* is a genus of *Prevotellaceae*, and the clinical significance of this genus remains unclear. Decreased inflammatory cytokine expression in the mouse intestine following interferon tau (IFNT) supplementation led to increased *Alloprevotella* abundance in the colon (Ren et al., 2016), indicating that *Alloprevotella* has positive effects on the intestinal mucosa. Intriguingly, the abundance of *Alloprevotella* and *Bacteroides* was distinctly reduced in the TLR4 KO group, indicating a complex relationship among TLR4, the microbiota, and intestinal immunity.

Notably, at the genus level, VA and TLR4 had a combined effect on the abundance of *Allobaculum* (*Firmicutes*), *Ruminiclostridium_9* (*Firmicutes*), *Alistipes* (*Bacteroidetes*), and *Rikenellaceae_RC9* (*Bacteroidetes*). *Allobaculum* has been shown to prevent dextran sulfate sodium (DSS)-induced inflammation (Wang et al., 2015), and the abundance of this genus was seen to be positively correlated with markers of ileal immunity (Cox et al., 2014). However, in the present study, the abundance of *Allobaculum* in the WT VAD group was significantly higher than that in the WT VAN group, but there was no difference in the abundance of this genus between the VAN and VAD groups of TLR4 KO mice, suggesting that VA might regulate *Allobaculum* via TLR4. *Ruminiclostridium_9* belongs to the *Ruminococcaceae* family and is associated with the release of inflammatory and cytotoxic factors from the gut for maintenance of a stable intestinal microecology (Cheng et al., 2017; Ma et al., 2017; Wu et al., 2017). Although there were no differences among the WT VAN, WT VAD, and KO VAD groups in terms of the abundance of *Ruminiclostridium_9*, we found that the *Ruminiclostridium_9* abundance in the KO VAN group was significantly higher than that in the other three groups. These results suggest that TLR4 may be involved in the regulation of intestinal microbiota by VA, but the specific mechanism of regulation and the clinical significance of this regulation need to be further explored. A cross-study analysis showed that *Alistipes* species are associated with healthy subjects rather than with individuals with gut disease (Mancabelli et al., 2017). In a murine model of DSS-induced colitis, *Alistipes finegoldii (Alistipes)* was seen to be protective against colitis (Dziarski et al., 2016). However, the *Alistipes* genus was found to be associated with colorectal cancer, and the abundance of this genus exhibited a negative correlation with the consumption of fruits and vegetables (Feng et al., 2015; Dai et al., 2018). *Rikenellaceae_RC9*, similar to *Alistipes*, belongs to the *Rikenellaceae* family. The abundance of both these genera was affected by VA and TLR4. However, the specific regulatory mechanisms remain unclear.

A large number of studies have shown that the VA nutritional state affects the abundance and composition of the intestinal microbiota (Amit-Romach et al., 2009; Hibberd et al., 2017). There is also considerable variation and discrepancy associated with identification of bacterial markers of VAD among different studies (Chiodini et al., 2015; Liu et al., 2016) and different disease models, such as models of persistent diarrhea and necrotizing enterocolitis (Lv et al., 2016; Xiao et al., 2018). On the other hand, the intestinal microflora was shown to affect the bioavailability of dietary α- and β-carotene and the activity of VA in rats (Grolier et al., 1998). These results indicate that VA levels and the intestinal microbiota are interrelated. Many studies have shown that VAD impairs GI mucosal barrier integrity by altering bacterial populations, the expression of innate immunity-related genes and the number of immune cells (Amit-Romach et al., 2009; Liu et al., 2014; Li et al., 2017). Our study further demonstrated the independent effects of VA and TLR4 on intestinal mucosa-related bacteria. Our study is the first to indicate that TLR4 is involved in regulation of the colonic mucosal microbiota by VA, providing a foundation for elucidating the relationships among VA, the intestinal microecology and intestinal innate immunity. This result further shows that VA regulates TLR4 to improve intestinal barrier function, as described in our previous study (Li et al., 2017). However, elucidation of the specific regulatory mechanism remains challenging and requires further study and the development of novel approaches.

## Conclusion

VAD decreased the abundance of *Anaerotruncus* (*Firmicutes*), *Oscillibacter* (*Firmicutes*), *Lachnospiraceae _NK4A136 _group* (*Firmicutes*), and *Mucispirillum* (*Deferribacteres*) and increased the abundance of *Parasutterella* (*Proteobacteria*). TLR4 KO decreased the abundance of *Bacteroides* (*Bacteroidetes*) and *Alloprevotella* (*Bacteroidetes*). However, the abundance of *Allobaculum* (*Firmicutes*), *Ruminiclostridium_9* (*Firmicutes*), *Alistipes* (*Bacteroidetes*), and *Rikenellaceae_RC9* (*Bacteroidetes*) impacted the interaction between VA and TLR4. Therefore, TLR4 may play a pivotal role in the regulation of the intestinal mucosa-associated microbiota and maintenance of the intestinal microecology mediated by VA.

## Author Contributions

LX performed the experiments and analyzed the data. BC and DF assisted in completion of the experiments. TY provided technical guidance regarding the use of HPLC. LX and JC wrote the manuscript. TL and JC designed the study. JC provided financial support for the study. All authors read and approved the final manuscript.

## Conflict of Interest Statement

Our team bears a patent (Patent No. ZL201010233032.8) on the formula of the vitamin A normal and vitamin A deficiency animal feed in China.

## Footnotes

1 http://drive5.com/uparse/

2 http://rdp.cme.msu.edu/

3 http://drive5.com/uparse/

## Abbreviations

HPLC: high-performance liquid chromatography
IBD: inflammatory bowel disease
Mean ± SEM: mean ± standard error of mean
OTU: operational taxonomic units
PBS: phosphate-buffered saline
PCR: polymerase chain reaction
RARβ: retinoic acid receptor β
TLR4^-/-^: Toll-like receptor 4 knockout
TLR4: Toll-like receptor 4
VA: vitamin A
VAD: vitamin A deficiency
VAN: vitamin A normal
WT: wild-type
ZO-2: zonula-occludens 2
